# A Review of Food-Related Social Media and Its Relationship to Body Image and Disordered Eating

**DOI:** 10.3390/nu17020342

**Published:** 2025-01-18

**Authors:** Bethany A. Roorda, Stephanie E. Cassin

**Affiliations:** Department of Psychology, Toronto Metropolitan University, 350 Victoria St., Toronto, ON M5B 2K3, Canada

**Keywords:** social media, mukbang, food blog, fitspiration, dieting, disordered eating, body image

## Abstract

Background/Objectives: Appearance-related social media, such as “thinspiration” and “fitspiration” posts, have been shown to contribute to poor body image and disordered eating. Food-related social media is becoming increasingly popular; however, far less is known about its relationship to body image and disordered eating. Methods: The current review searched PsycNet and PubMed (Medline) for all the literature examining food-related social media and its relationship with body image and/or disordered eating outcomes. Results: From 796 initial hits, the search identified 16 relevant studies. The study designs and types of media examined varied widely, including mukbang videos, food blogs, and “What I Eat In A Day” videos. Findings on the relationship between food-related social media and outcome variables were quite mixed, perhaps speaking to the wide variety of media included in the review. Conclusions: The existing literature is sparce, but overall, it suggests a potential relationship between food-related social media, negative body image, and disordered eating. Additional experimental research is needed to clarify outcomes for different media types (e.g., food blogs versus mukbang videos) and to determine the direction of causality for each.

## 1. Introduction

Many individuals in the Western world, particularly women, experience difficulties accepting and appreciating their own bodies. Weight dissatisfaction has been well documented across decades; to such an extent, in fact, that it has been called “normative discontent” [1]. Frederick and colleagues [2], in their chapter on the epidemiology of body dissatisfaction, estimate that 30% of women experience body dissatisfaction, while a systematic review [3] estimates body dissatisfaction in women to range from 11% to 72% in the United States, depending on the measurements used (e.g., 11% are dissatisfied with their face; 72% reported that they are less than “very satisfied” with their appearance). Such negative body image is associated with numerous adverse outcomes, including poor self-esteem [4], social anxiety [5], and eating disorders [6]. A phenomenon this widespread demands research into both the etiology and treatment of body dissatisfaction in order to mitigate the detrimental effects.

Sociocultural theory speculates that media is one of the main sources of body dissatisfaction [7]. Over the past decade, these principles have been studied in the context of bodies viewed via social media (e.g., Instagram, Facebook, Pinterest). One meta-analysis [8] examined the relationship between body image and “social media use” (pg. 261) in 56 cross-sectional studies and found that more exposure to social media was significantly related to negative body image, though the effect size was small overall. Recent correlational and experimental studies support this relationship (e.g., [9,10]). For example, one experiment found that females who looked at photos of attractive female social media influencers experienced poorer body image than females who looked at nature images, even when the popularity of the social media account was low [11].

“Thinspiration” and “fitspiration” are two appearance-related movements within the social media sphere that may be particularly harmful to viewers’ body satisfaction. Thinspiration refers to social media posted with the intent to motivate women to be thin [12], which may include photos of thin women [13] and/or problematic messaging such as “Every time you say no to food, you say yes to thin” ([14], p. 203). Fitspiration is a similar phenomenon, with a focus on looking and feeling “fit” rather than thin; however, some argue that “fitspiration” still promotes a very specific (and typically still thin) body ideal [14]. Thinspiration and fitspiration media can include messaging about weight loss, celebrating thinness, guilt about one’s appearance, objectifying bodies, weight stigma, and diets, albeit at different rates between the two types of media [13].

Both thinspiration and fitspiration have largely been found to contribute to poor body image. For example, one experiment showed female undergraduate participants thinspiration, fitspiration, or travel (control) images from Instagram, and found that participants experienced less body satisfaction after exposure to the thinspiration and fitspiration photos than after exposure to the control photos [14]. A similar pattern has been replicated while investigating the impact of fitspiration photos on body dissatisfaction [15,16].

Given the close relationship between body image and eating disorders and the negative impact of media ideals and appearance-related social media on body image, it is perhaps not surprising that media may impact disordered eating symptoms as well. For example, Dakanalis and colleagues [17] suggest that the adolescents’ internalization of media thin ideals is related to disordered eating through self-objectification and shame about one’s own body. Walker and colleagues [18] found making comparisons to others’ bodies when using Facebook to be associated with symptoms of eating pathology, and Griffiths and colleagues [19] found that viewing thinspiration and fitspiration is related to eating pathology amongst adults with eating disorders [19]. These findings indicate that eating disorder symptomology may be related to social media use, perhaps indirectly through body image-related variables.

Food-related media is another phenomenon that has migrated to the social media sphere. Food-related social media includes a variety of media postings, such as blog posts written about food, videos of what an individual eats at a given time, Mukbang videos (a video genre where an individual films themselves eating an exorbitant amount of food in one sitting [20]); or even tantalizing images of appetizing food (called “food porn” [21]). These types of media are extremely popular. Videos on video-heavy social media sites that track what one eats in a day (i.e., “What I Eat In A Day” videos) are so widespread that celebrities such as Kylie Jenner have posted these types of videos on TikTok with over 100 million views. Some popular mukbang channels on Youtube have millions of subscribers (for example, content creator @tzuyang has 6.81 million subscribers on Youtube). Mejova and colleagues [22] conducted an analysis of all Instagram posts that included the hashtag “foodporn” and found over 9 million results from just over 5 months, indicating this photo genre’s widespread popularity.

It is possible that these food and diet-related social media pages, videos, and posts may affect the body image and/or eating habits of those viewing them. Carrote and colleagues [23] conducted a study to examine various correlates of interacting with “health and fitness-related social media” (p. 5). They found that 32% of individuals who self-reported having an eating disorder followed “detox” social media (media promoting diets that aim to “detoxify” the body), compared to only 13% of individuals without an eating disorder. Jin [24] found that having symptoms of either anorexia nervosa or bulimia nervosa seems to influence an individuals’ reaction to food-related social media. For example, those with eating disorder symptoms had higher “social media envy” versus those without eating disorder symptoms when a thin foodie posted food pictures, whereas levels of envy were similar when the food pictures were posted by someone with a larger body. These studies suggest that there may be an association between eating disorders and food-related social media, although they do not point to the direct impact of this type of media on eating disorder symptoms.

A recent systematic review [25] examined the relationship between food-related social media and body image/disordered eating outcomes. Their investigation suggests a link between these variables; however, the review also included studies that assessed body image or eating-adjacent variables that may not necessarily be indicative of body image or eating issues. For example, Wu and colleagues included studies whose eating or body-related outcome was hunger. Although hunger is eating-related, it is not necessarily related to *disordered* eating (i.e., there are many reasons why hunger levels could be lower or higher, and conclusions about disordered eating cannot be drawn without further investigation). The current review, therefore, aims to build upon Wu and colleagues’ work by updating the search given the proliferation of food-related social media over the past few years and refining the scope of the investigation by specifically examining the link between food-related social media and body image, disordered eating, and closely related constructs.

## 2. Methods

To gather published research examining the relationship between food-related social media and body image and/or eating disorder symptoms, PsycNet databases were searched on 18 May 2022 and the PubMed (Medline) database was searched on 26 May 2022 and updated on 28 May 2024. Search terms for all databases included food-related terms (i.e., “food-related” OR food OR eat), social media-related terms (i.e., “social media” OR TikTok OR Instagram OR Facebook OR YouTube), and body image/eating disorder-related terms (i.e., “body image” OR “body satisfaction” OR “body dissatisfaction” OR “body image flexibility” OR “thin-ideal” OR “weight bias” OR “weight stigma” OR “eating disorder” OR “disordered eating” OR “eating symptoms” OR “restriction” OR “binge” OR “binging” OR “purging” OR “purge” OR “exercise” OR “anorexia” OR “bulimia” OR “binge eating disorder”). There were no year restrictions set for these searches. A search for other relevant articles was conducted by reading through titles on the reference lists of each of the publications included in the current review, as well as the systematic review recently published by Wu and colleagues [25].

Titles and abstracts were then screened for study inclusion by the first author. Inclusion criteria included any paper that: (a) discussed social media that was food-related in some way (e.g., photos of food; videos of someone eating), and (b) examined body image and/or disordered eating constructs, including eating disorder symptoms. Exclusion criteria included: (a) reviews or meta-analyses, (b) studies in languages other than English, and (c) studies that did not contain numerical data.

**Figure 1:**
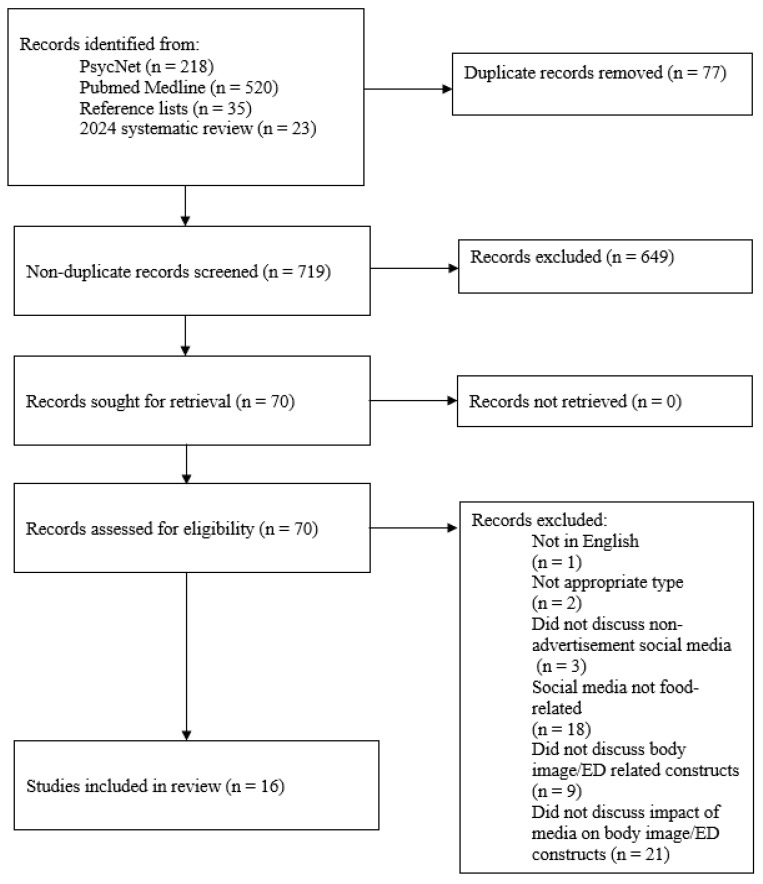
Summary of the review search and selection process.

All studies remaining after the Title/Abstract screen were then screened by the first author at the full text level based on the same inclusion/exclusion standards as above to ensure that each study met the review criteria. Data were then manually extracted by the first author. See Figure 1 for a summary of the search and selection process.

## 3. Results

A total of sixteen relevant studies were identified via these searches and subsequently included in the review. Two studies were qualitative, three were correlational, six were quasi-experimental, and five were experimental. Studies were conducted in a variety of countries, mostly Western (e.g., USA, Canada, Australia) or Middle Eastern (e.g., Turkey, Saudi Arabia). Participant demographics varied, but most studies had young adult participants, and many had a majority of (or all) participants identifying as female. Most studies conducted in Western countries reported that a majority of participants identified as white. Social media content examined in the studies also varied widely; many examined mukbangs, clean eating content, nutrition advice, or photos/videos of food. The summary of study characteristics and results is presented in Table 1.

### 3.1. Qualitative Studies

A qualitative study by Bissonnette-Maheux and colleagues [26] examined women’s focus group conversations after having browsed four specific dietician-authored blogs about nutrition and “healthy eating.” They reported that two of their six focus groups discussed experiencing guilt about their own eating habits after having read the blogs.

A more recent qualitative study by Strand and Gustafsson [20] focused on mukbang videos. They identified a number of themes from comments on mukbang videos posted on Youtube and Reddit [20]. Two themes relevant to the current review pertained to the impact of watching mukbang videos on the viewers’ own disordered eating behaviours [20]. One-hundred-and-fifty individual comments (out of 1316 total comments; 11.4%) reported that eating habits became more restrictive after viewing a mukbang, whereas 75 (5.7%) reported the opposite (i.e., overeating [20]).

### 3.2. Correlational Studies

von Ash and colleagues [27] conducted a correlational study examining mukbang viewing-related variables in a diverse sample of adults. They found mixed evidence regarding the relationship between self-reported frequency of viewing mukbang videos, time spent viewing mukbang videos, and “addiction” to viewing mukbang videos to body image and eating disorder variables. After adjusting for demographic variables, all mukbang variables were related to body dissatisfaction; frequency was positively related, whereas time spent and “addiction” to viewing mukbang videos were negatively related to body dissatisfaction. Mukbang viewing frequency was negatively related to one symptom of purging (overexercise) and “addiction” was related to various symptoms of binging and purging, but time spent watching mukbang videos was not related to these symptoms. Conversely, time spent viewing mukbang videos was the only variable related to overevaluation of shape and weight, and it was related in a positive direction (greater viewing time associated with greater overevaluation). All effect sizes were small. Interestingly, they found that none of these variables were related to dietary restraint. Kircaburun and colleagues [28] similarly examined mukbang “addiction” amongst young adults in Turkey and found it to be positively associated with eating disorder symptoms.

Another correlational study examined how other (i.e., non-mukbang) food/diet-related social media posts may relate to body image and eating outcomes. Wu and colleagues [29] examined posting and viewing clean eating Instagram content and compared it to posting and viewing fitspiration content. Engagement with these two types of content were similarly related to body image and disordered eating variables amongst women in Australia. Specifically, posting these types of content was related to greater compulsive exercise and athletic ideal internalization, but not thin-ideal internalization, body dissatisfaction, drive for thinness, or symptoms of bulimia nervosa, with small to medium effect sizes. Merely viewing these types of media was related to all these outcomes, also with small to medium effect sizes.

### 3.3. Quasi-Experimental Studies

The six quasi-experimental studies identified by this review were all cross-sectional and examined a variety of topics. One by Jeong and colleagues [30] examined mukbang watching, and found that amongst adolescents in South Korea, watching mukbangs every day (or “cookbangs;” i.e., videos of someone cooking a large quantity of food) seemed to be related to how one perceived their own body shape (i.e., “body shape perception”). However, they found that the existence and direction of these relationships differed according to the adolescent’s gender; boys tended to perceive their own bodies as being smaller than they were, whereas girls tended to perceive their own bodies as larger than they were. Boys were found to have body image distortion at higher frequencies of mukbang viewing, whereas this was not true for girls. Notably, these results seemed to be consistent with the general direction of girls and boys regardless of mukbang viewing (e.g., even amongst boys who reported no mukbang viewing, more boys reported believing their bodies were smaller than they were compared to larger). More descriptively, this study also reported relationships between mukbang/cookbang viewing and participants’ eating patterns. A greater percentage of boys and girls who viewed mukbang videos daily reported an impact on their eating behaviours in comparison to those who rarely viewed mukbang videos.

Another quasi-experimental study by Tazeoglu and Kuyulu Bozdogan [31] found that amongst a sample of adults in Turkey, a significantly greater percentage of women (72.8%) than men (38.9%) endorsed eating-related guilt when they ate directly after watching a food-related video on social media.

Caner and colleagues [32] studied outcomes related to viewing food/diet-related content shared by social media influencers in a sample of 1363 adolescents in Turkey. They asked students to complete a survey that included questions about what types of influencers the adolescents followed on social media. They found that following food/diet influencers was related to more emotional eating and social appearance anxiety than following other types of influencers (even exercise-related influencers).

A study conducted by Allen and colleagues [33] examined the relationship between food-related social media and body image/eating disorder symptomatology amongst 762 women in Australia. These researchers specifically examined “clean eating blogs;” that is, blog posts that promote generally low-sugar, low-carb, and “whole” food diets. The study found that individuals who reported either regularly or rarely following the guidance of these types of blogs reported higher dietary restraint than individuals who never followed these blogs’ tips. Further, those who regularly followed the advice of clean eating bloggers also reported higher dietary restraint than those who rarely did so.

Al-Bisher and Al-Otaibi [34] examined the relationship between “eating concerns” and the use of social media to obtain information about nutrition/diet amongst adult women in Saudi Arabia. The researchers found a positive relationship between these variables such that those who spent between 30 and 60 min actively searching for nutrition-related content on social media, those who reported that dieticians on social media were their preferred way to learn about nutrition, and those who were more interested in social media related to dieting and dietary supplements were all more likely to have eating concerns, all with large effect sizes [34]. Abu Alwafa and Badrasawi [35] similarly discovered that body appreciation was negatively related to nutrition-related social media amongst Palestinian women, specifically, following the food-related guidance on celebrity social media pages.

**Table 1:** Review results summary.

Author, Year (Country)	Study Design	Participant Characteristics	Media Type	Outcome(s)	Overall Results
Bissonnette-Maheux et al., 2015 [26] (Canada)	Qualitative	*n*= 33; mean age = 44; 100% female; 100% white; 30% family income between $20,000–$49,999	Healthy eating blogs, written by registered dietician	NA	Two of six groups discussed guilt about eating habits after having read the blogs.
Strand and Gustafsson, 2020 [20] (Sweden)	Qualitative	*n* = 1316 social media comments	Mukbang videos	NA	A total of 150 out of 1316 (11.4%) comments discussed increased dietary restriction; 75/1316 (5.7%) discussed overeating.
von Ash et al., 2023 [27] (USA)	Correlational	*n* = 264; mean age = 33.7; 49% female; 40% white	Mukbang videos (viewing frequency, time spent viewing, mukbang addiction)	Body dissatisfaction; shape/weight overevaluation; dietary restraint	Viewing frequency (*β* = 0.11, f^2^ = 0.02), time spent viewing (*β* = −2.05, f^2^ = 0.02) and addiction (*β* = −1.28, f^2^ = 0.08) related to body dissatisfaction. Viewing frequency (*β* = −0.33) and addiction (*β*s ≥ 0.28, 0.03 ≤ f^2^ ≥ 0.13) related to various binging and/or purging symptoms; time spent viewing related to overevaluation of shape and weight (*β* = 2.77, f^2^ = 0.06). Correlations between other variables not significant.
Kircaburun et al., 2021 [28] (Turkey)	Correlational	*n* = 140; mean age = 21.7; 66% female	Mukbang videos	Disordered eating symptomology	Mukbang addiction related to more symptoms of disordered eating (*β* = 0.43, *r* = 0.24, *p* < 0.01).
Jeong et al., 2024 [30] (South Korea)	Quasi-experimental	*n* = 50,455; 100% adolescents; 49% female; generally middle class	Mukbang and cookbang videos	Eating behaviors; body shape perception; body image distortion	Boys viewing mukbangs each day tended to believe their bodies were smaller, while girls tended to believe bodies were larger. More boys found to have body image distortion at higher mukbang viewing frequencies.
Wu et al., 2022 [29] (Australia)	Correlational	*n* = 269; mean age = 20.7; 100% women; 84.8% white	Clean eating content on Instagram	Compulsive exercise; athletic-ideal internalization; thin-ideal internalization; drive for thinness; disordered eating symptomology; body dissatisfaction	Posting related to compulsive exercise (*r*s ≥ 0.24) and athletic ideal internalization (*r*s ≥ 0.24), but not thin-ideal internalization, body dissatisfaction, drive for thinness, or bulimia nervosa symptoms (*r*s ≤ 0.10). Viewing related to all outcomes (*r*s ≥ 0.13).
Allen et al., 2018 [33] (Australia)	Quasi-experimental	*n* = 762; median age = 27; 100% female; 87% white	Clean eating blogs	Dietary restraint	Individuals following guidance of blogs reported higher dietary restraint than individuals who did not (*F*(2729) = 26.93; *p* ≤ 0.01).
Al-Bisher and Al-Otaibi, 2022 [34] (Saudi Arabia)	Quasi-Experimental	*n* = 1092; mean age = 23; 100% female	Nutrition information on various social media sites	Eating concerns	Those who: spent 30–60 min searching for nutrition content on social media (*OR* = 1.09; *p* = ≤ 0.05) preferred to learn about nutrition through dieticians on social media (*OR* = 1.17; *p* ≤ 0.001) or were more interested in social media related to dieting/dietary supplements (*OR* = 0.87; *p* < 0.001) and had a higher likelihood of eating concerns.
Abu Alwafa and Badrasawi, 2023 [35] (Palestine)	Quasi-Experimental	*n* = 905; mean age = 20; 100% female	Nutrition advice from model and celebrity social media accounts	Body appreciation	Body appreciation negatively related to taking the food-related guidance on celebrity social media channels (*ES* = 0.23, *p* = 0.01).
Caner et al., 2022 [32] (Turkey)	Quasi-Experimental	*n* = 1363; mean age = 15.9; 63.8% girls; 63.4% perceived “normal” family income	Nutrition/diet content on influencer accounts	Emotional eating	Following these influencers was related to more emotional eating and social appearance anxiety than following other types of influencers (Kruskal–Wallis H Test value ≤ 10.66, *p*s ≤ 0.03).
Tazeoglu and Kuyulu Bozdogan, 2022 [31] (Turkey)	Quasi-Experimental	*n* = 1160; mean age = 21.8; 52.2% female	Food videos	Eating-related guilt	More women (72.8%) than men (38.9%) said that they feel guilty about eating something directly following watching a video (*p* = 0.01).
Kinkel-Ram et al., 2022 [36] (USA)	Experimental	Sample 1: *n* = 222; mean age = 18.9; 100% women; 85.9% white. Sample 2: *n* = 214; mean age = 19.3; 100% female; 71.1% white	Photos of low-calorie food	Body image; intent for disordered eating	Viewing low-calorie food photos promoted more intention for disordered eating than viewing travel photos amongst women in midwestern US sample: *F*(1217) = 323.37, *p* < 0.001, *partial η*^2^ = 0.03). Neither sample demonstrated differences in body image across the two conditions (*p*s > 0.15).
Zeeni et al., 2024 [37] (Lebanon)	Experimental	*n* = 63; mean age = 22; 68.3% female	“Junk” food Instagram photos	Food craving	Browsing photos on Instagram resulted in greater food craving for salty, savoury, and fatty foods (*t*s ≥ 2.93, *p*s ≤ 0.005, 0.47 ≥ *d*s ≤ 0.70) than browsing control accounts. No differences in body image (*t* = 1.38, *p* = 0.17, *d* = 0.18) or cravings for sweet foods (*t* =2.0, *p* = 0.052, *d* = 0.29).
Fiuza and Rodgers, 2023 [38] (USA)	Experimental	*n* = 421; mean age = 19.5; 100% women	Diet culture-related TikTok videos (for or against diet culture)	Weight/shape satisfaction; intuitive eating; body appreciation; restriction; exercise urges	Diet video condition promoted lower weight and shape satisfaction compared to condition against diet culture (*F*(2, 392) = 5.05, *p =* 0.01, *partial η*^2^ = 0.03). Condition against diet culture promoted more body appreciation compared to neutral video condition (*F*(2, 393) = 3.09, *p =* 0.05, *partial η*^2^ = 0.02). Video against diet culture promoted more intuitive eating compared to the neutral video condition (*F*(2, 392) = 4.29, *p =* 0.01, *partial η*^2^ = 0.02). No significant differences amongst conditions for restriction or exercise urge scores.
Drivas et al., 2024 [39] (USA)	Experimental	*n* = 316; mean age = 19.8; 62.7% women; 75.6% white	“What I Eat in a Day” videos	Body appreciation; body satisfaction; diet intentions	Video type (i.e., viewing higher calorie days) indirectly and positively impacted body appreciation and diet intentions, indirectly negatively impacted body dissatisfaction.
Neter et al., 2018 [40] (Israel)	Experimental	*n* = 165; mean age = 25.7; 84.8% female	Food porn featured in a travel vlog	Food cravings	Those who viewed vlogs with food porn did not report different levels of craving than those who watched travel vlogs without food porn (*F*(1162) = 0.77; *p* = 0.38).

### 3.4. Experimental Studies

Two of the five experimental studies identified during the search examined food-related posts on Instagram. Kinkel-Ram and colleagues [36] examined the relationship between food-related social media and body image/disordered eating intentions in two samples of women in the USA. Overall, they found that viewing low-calorie food photos (e.g., vegetables) promoted more intention for disordered eating than viewing travel photos (control; *partial η*^2^ = 0.03) amongst women in sample 1 only, with a small effect size. However, neither sample demonstrated differences in body image across the two conditions.

Zeeni and colleagues [37] similarly presented study participants in Lebanon with either food-related Instagram photos (specifically “junk food” photos) or control Instagram images. These researchers found that browsing “junk food” Instagram accounts resulted in greater food craving than browsing the control accounts, with overall medium effect sizes, but those in the junk food condition and control condition reported similar levels of state body image [37].

Two of the experimental studies examined TikTok content. Fiuza and Rodgers [38] examined body appreciation and disordered eating symptoms in women in the USA after watching diet-culture related videos (i.e., What I Eat in A Day videos with approximately 1500 kcal or less pictured as daily intake) on TikTok compared to TikTok videos whose content was clearly against diet culture (i.e., videos speaking against restricting certain foods or exercising for weight loss reasons only). The diet-promoting videos seemed to adversely impact weight and shape satisfaction, whereas the videos against diet culture promoted body appreciation and intuitive eating, all with small effect sizes [38]. They found no significant differences in video condition when comparing urges to engage in dietary restriction and exercise.

Drivas and colleagues [39] examined the impact of watching “What I Eat In A Day” TikTok videos on adults in the USA. Participants were randomized to view videos in which someone consumed more versus fewer calories (operationalized in a pilot study as “diets [that]… would lead to weight loss” or “weight gain”, p. 5) throughout the day. These researchers did not examine the direct effect of video type on body image or eating variables but rather tested a model that examined the indirect effect of video type on body image and dieting through social comparison variables and affect. They found that video type (i.e., viewing higher calorie days) indirectly and positively impacted body appreciation and diet intentions, and indirectly and negatively impacted body dissatisfaction (i.e., less body dissatisfaction) [39].

Neter and colleagues [40] conducted a study examining the impact of “food porn” on adults in Israel. They found that those who viewed “food porn” images as part of a travel vlog did not report different levels of food craving compared to those who watched more neutral travel vlogs.

## 4. Discussion

The body of literature examining the relationship between food-related social media and body image and eating disorder related variables is currently in its infancy. This review identified 16 articles that fit into this category, only five of which were experimental studies. The two qualitative studies suggested a relationship between food-related social media and disordered eating habits or emotions. Strand and Gustafsson [20] found in their qualitative study that mukbang videos influenced some individuals’ disordered eating habits (both restrictive and overeating), and Bissonnette-Maheux and colleagues [26] found healthy eating blogs to promote guilt about eating habits amongst some women.

Research examining the influence of mukbangs have generated somewhat mixed results. Although greater mukbang viewing generally seemed to be related to poorer body image and/or disordered eating, von Ash and colleagues found some unexpected results such that various mukbang-viewing-related measures were differently related to certain body image and disordered eating variables [27]. This finding indicates the importance of examining various aspects of social media watching, as watching for 30 min on one occasion per week may have a different impact than 2 min of viewing multiple times per week.

Jeong and colleagues [30] found that watching mukbangs and cookbangs seemed to be related to how both girls and boys perceived their body shapes. However, it is important to note that the direction of these relationships was similar regardless of mukbang/cookbang viewing. They may, therefore, be due to the general body perceptions of boys and girls in South Korea in general and simply be amplified upon viewing of mukbang/cookbang videos.

Importantly, Strand and Gustafsson’s [20] qualitative study into mukbang comments also found that themes related to disordered eating arose in the comments of these videos. However, they examined only English comments despite mukbang videos originally being a South Korean phenomenon, with four out of the five videos being Korean or Japanese in origin, and a large number of the posted comments being non-English. Due to this limitation, they may have missed a large amount of information that may be relevant to the relationship between mukbang videos and disordered eating symptoms [20].

The correlational and quasi-experimental studies overall found relationships between food-related social media and disordered eating/poor body image. This seems to be especially true for *consuming* these types of social media, as most studies focused on viewing social media as opposed to posting. Wu and colleagues [29] found that posting clean eating media was not related to thin-ideal internalization, body dissatisfaction, drive for thinness, or symptoms of bulimia nervosa, though it was related to other disordered eating and body image-related variables. As this is the only study that has thus far examined the outcomes related to posting food-related content, these results must be taken as preliminary and require future replication with various types of food-related media. However, taken together, these correlational studies suggest a relationship between food-related social media, disordered eating behaviour, and body image across various types of media. These studies are not experimental and, therefore, do not imply causation. It is important to note that individuals who already engage in disordered eating or who have poor body image may be attracted to these types of food-related media.

The experimental studies published on this topic provide mixed results. For example, Kinkel-Ram and colleagues [36] found that low-calorie food photos promoted more intention for disordered eating than control photos in one of their two samples, but the photos did not impact body image. Similarly, viewing “junk food” photos did not impact body image, but did impact craving levels [37]. Conversely, viewing food porn did not impact craving levels at all [40]. Women who watched diet videos did not report worse body image than a control group, but they did report poorer body image than women who watched anti-diet videos. Similarly, women who watched anti-diet videos reported more body appreciation than the control group (but not more than those who watched the diet videos) [38]. These anti-diet videos also promoted more intuitive eating than the control group, but no group differences were found for other disordered eating variables [38]. Meanwhile, higher calorie “What I Eat in a Day” videos seem to promote both greater body appreciation and a greater intention to diet, which seems contradictory to others’ findings [39]. These mixed results may be due in part to the various types of food-related media featured in these studies—perhaps content that is more diet-related (e.g., low-calorie food photos/videos) versus non-diet related (e.g., “food porn” images) may be more likely to promote disordered eating or poor body image. As each of these media types has only been investigated in one experiment each, further investigation into the outcomes of viewing various types of food-and diet-related media is warranted before any conclusions are drawn.

Due to the heterogeneity of food-related social media and the mixed results, it is difficult to discern the practical implications of the research conducted to date for mitigating poor body image and disordered eating. However, some preliminary results [36,39] suggest a relationship between at least some types of food related social media (low calorie food photos, high calorie “What I Eat In A Day” videos) and intention for disordered eating. This may indicate a need for individuals engaging with social media to be aware of the impact the videos they watch may have on their eating intentions. Although these studies did not focus on individuals with eating disorders specifically, it may follow that those with eating disorders would benefit from awareness about the impact of consumed media content on their symptoms. Including psychoeducation into meetings with dieticians or in psychotherapy may be beneficial for such individuals. Additionally, results from the study by Fiuza and Rodgers [38] suggest that purposefully exposing individuals to anti-diet videos, and/or encouraging individuals to engage with such content on a regular basis, may be helpful in boosting body image overall. 

Overall, the limited research conducted to date suggests a potential relationship between food-related social media (both the food content and the images of individuals posting the food content), body image, and disordered eating. However, the heterogeneity across studies regarding participant samples, research designs, type of food-related social media, and outcomes assessed preclude drawing conclusions about the specific impact. The heterogeneity in the type of food-related social media examined across studies is to be expected considering the dynamic nature of content that is trending on social media. The outcomes examined to date have been limited to immediate self-report measures (e.g., intention to diet, intuitive eating), and it would be beneficial to assess the impact on eating disorder symptoms, eating patterns, and body image over days or weeks after viewing food-related social media. A qualitative exploration of the comments posted in response to food-related social media may also provide some insights into the impact of the videos. This would give in-depth and detailed information about immediate reactions to media that quantitative methods often miss. Finally, it would be informative to learn whether the impact of food-related social media differs depending on whether an individual actively seeks it out (i.e., searching for and clicking on the content) or passively views it (i.e., scrolling through a social media feed); perhaps those who are more intentional about food media consumption may find themselves more actively intending to change their own eating in response to the media.

Additional future research may include uncovering the mechanisms that drive the link between food-related social media and negative body- and eating-related outcomes. Research into appearance-related social media has drawn heavily on Social Comparison Theory [41], and many studies examine the link between viewing appearance-related media, comparison, and body image (e.g., [42,43]). Drivas and colleagues [39] have started to investigate whether such a relationship exists for food-related social media as well, and it would be interesting to continue to examine the strength of this relationship in this newer realm of research. Overall, further investigation into food-related social media and its impact on body image and eating is warranted considering the abundance of food-related social media and the plethora of negative consequences of experiencing poor body image and disordered eating.

## References

[1] Rodin J., Siberstein L., Striegel-Moore R. (1984). Women and weight: A normative discontent. Neb. Symp. Motiv..

[2] Frederick D.A., Jafary A.M., Gruys K., Daniels E.A., Cash T.F. (2012). Surveys and the Epidemiology of Body Image Dissatisfaction. Encyclopedia of Body Image and Human Appearance.

[3] Fiske L., Fallon E.A., Redding C.A. (2014). Prevalence of body dissatisfaction among United States adults: Review and recommendations for future research. Eat. Behav..

[4] Brechan I., Kvalem I.L. (2015). Relationship between body dissatisfaction and disordered eating: Mediating role of self-esteem and depression. Eat. Behav..

[5] Pawijit Y., Likhitsuwan W., Ludington J., Pisitsungkagarn K. (2019). Looks can be deceiving: Body image dissatisfaction relates to social anxiety through fear of negative evaluation. IJAMH Int. J. Adolesc. Med. Health.

[6] Prnjak K., Hay P., Mond J., Bussey K., Trompeter N., Lonergan A., Mitchison D. (2021). The distinct role of body image aspects in predicting eating disorder onset in adolescents after one year. J. Abnorm. Psychol..

[7] Thompson J.K., Heinberg L.J., Altabe M., Tantleff-Dunn S. (1999). Exacting Beauty: Theory, Assessment, and Treatment of Body Image Disturbance.

[8] Saipoo A.N., Vahedi Z. (2019). A meta-analytic review of the relationship between social media use and body image disturbance. Comput. Hum. Behav..

[9] Hogue J.V., Mills J.S. (2019). The effects of active social media engagement with peers on body image in young women. Body Image.

[10] Markey C.H., August K.J., Gillen M.M., Rosenbaum D.L. (2024). An examination of youths’ social media use and body image: Considering TikTok, Snapchat, and Instagram. J. Media Psychol. Theor. Methods Appl..

[11] Lowe-Calverley E., Grieve R. (2021). Do the metrics matter? An experimental investigation of Instagram influencer effects no mood and body dissatisfaction. Body Image.

[12] Ghaznavi J., Taylor L.D. (2015). Bones, body parts, and sex appeal: An analysis of #thinspiration images on popular social media. Body Image.

[13] Boepple L., Thompson J.K. (2016). A content analytic comparison of fitspiration and thinspiration websites. Int. J. Eat. Disord..

[14] Dignard N.A.L., Jarry J.L. (2021). The “Little Red Riding Hood Effect:” Fitspiration is just as bad as thinspiration for women’s body satisfaction. Body Image.

[15] Ladwig G., Tanck J.A., Quittkat H.L., Vocks S. (2024). Risks and benefits of social media trends: The influence of “fitspiration”, “body positivity”, and text-based “body neutrality” on body dissatisfaction and affect in women with and without eating disorders. Body Image.

[16] Prichard I., Kavanagh E., Mulgrew K.E., Lim M.S.C., Tiggemann M. (2020). The effect of Instagram #fitspiration images on young women’s mood, body image, and exercise behaviour. Body Image.

[17] Dakanalis A., Carrà G., Calogero R., Fida R., Clerici M., Zanetti M.A., Riva G. (2015). The developmental effects of media-ideal internalization and self-objectification processes on adolescents negative body-feelings, dietary restraint, and binge eating. Eur. Child Adolesc. Psychiatry.

[18] Walker M., Thornton L., De Choudhury M., Teevan J., Bulik C.M., Levinson C.A., Zerwas S. (2015). Facebook use and disordered eating in college-aged women. J. Adolesc. Health.

[19] Griffiths S., Castle D., Cunningham M., Murray S.B., Bastian B., Barlow F.K. (2018). How does exposure to thinspiration and fitspiration relate to symptom severity among individuals with eating disorders? Evaluation of a proposed model. Body Image.

[20] Strand M., Gustafsson S.A. (2020). Mukbang and disordered eating: A netnographic analysis of online eating broadcasts. Cult. Med. Psychiatry.

[21] Versace F., Frank D.W., Stevens E.M., Deweese M.M., Guindani M., Schembre S.M. (2018). The reality of “food porn”: Larger brain responses to food-related cues than to erotic images predict cue-induced eating. Psychophysiol.

[22] Mejova Y., Abbar S., Haddadi H. Fetishizing food in digital age: #foodporn around the world. Proceedings of the International AAAI Conference on Web and Social Media.

[23] Carrote E.R., Vella A.M., Lim M.C.S. (2015). Predictors of “liking” three types of health and fitness-related content on social media: A cross-sectional study. J. Med. Internet Res..

[24] Jin S.V. (2018). Interactive effects of Instagram foodies’ hastagged #foodporn and peer users’ eating disorder on eating intention, envy, parasocial interaction, and online friendships. Cyberpsychol. Behav. Soc. Netw. Index.

[25] Wu Y., Kemps E., Prichard I. (2024). Digging into digital buffets: A systematic review of eating-related social media content and its relationship with body image and eating behaviours. Body Image.

[26] Bissonnette-Maheux V., Provencher V., Lapointe A., Dugrenier M., Dumas A.A., Pluye P., Straus S., Gagnon M.P., Desroches S. (2015). Exploring women’s beliefs and perceptions about healthy eating blogs: A qualitative study. J. Med. Internet Res..

[27] von Ash T., Huynh R., Deng C., White M.A. (2022). Associations between mukbang viewing and disordered eating behaviors. Int. J. Eat. Disord..

[28] Kircaburun K., Yurdagul C., Kuss D., Emirtekin E., Griffiths M.D. (2021). Problematic mukbang watching and its relationship to disordered eating and internet addiction: A pilot study among emerging adult mukbang watchers. Int. J. Ment. Health.

[29] Wu Y., Harford J., Petersen J., Prichard I. (2022). “Eat clean, train mean, get lean”: Body image and health behaviours of women who engage with fitspiration and clean eating imagery on Instagram. Body Image.

[30] Jeong H., Lee E., Han G. (2024). Association between mukbang and cookbang viewing and body image perception and BMI in adolescents. JHPN J. Health Popul. Nutr..

[31] Tazeoglu A., Kuyulu Bozdogan F.B. (2022). The effect of watching food videos on social media on increased appetite and food consumption. Nutr. Clin. Diet. Hosp..

[32] Caner N., Sezer Efe Y., Başdaş Ö. (2022). The contribution of social media addiction to adolescent LIFE: Social appearance anxiety. Curr. Psychol..

[33] Allen M., Dickinson K.M., Prichard I. (2018). The dirt on clean eating: A cross sectional analysis of dietary intake, restrained eating and opinions about clean eating among women. Nutrients.

[34] Al-Bisher M.M., Al-Otaibi H.H. (2022). Eating concerns associated with nutritional information obtained from social media among Saudi young females: A cross-sectional study. Int. J. Environ. Res. Public Health.

[35] Abu Alwafa R., Badrasawi M. (2023). Factors associated with positive body image among Palestinian university female students, cross-sectional study. Health Psychol. Behav..

[36] Kinkel-Ram S.S., Staples C., Rancourt D., Smith A.R. (2022). Food for thought: Examining the relationship between low calorie density foods in Instagram feeds and disordered eating symptoms among undergraduate women. Eat. Behav..

[37] Zeeni N., Abi Kharma J., Malli D., Khoury-Malhame M., Mattar L. (2024). Exposure to Instagram junk food content negatively impacts mood and cravings in young adults: A randomized controlled trial. Appetite.

[38] Fiuza A., Rodgers R.F. (2023). The effects of brief diet and anti-diet social media videos on body image and eating concerns among young women. Eat. Behav..

[39] Drivas M., Simone Reed O., Berndt-Goke M. (2024). #WhatIEatInADay: The effects of viewing food diary TikTok videos on young adults’ body image and intent to diet. Body Image.

[40] Neter E. (2018). The Effect of Exposure to Food in Social Networks on Food Cravings and External Eating. Doctoral Dissertation.

[41] Festinger L. (1954). A theory of social comparison process. Hum. Relat..

[42] Seekis V., Bradley G.L., Duffy A.L. (2020). Appearance-related social networking sites and body image in young women: Testing an objectification-social comparison model. PWQ Psychol. Women Q..

[43] Want S.C., Botres A., Vahedi Z., Middleton J.A. (2015). On the cognitive (in)efficiency of social comparisons with media images. Sex Roles.

